# Validation of the probability of survival using the triss methodology in the Spanish Trauma ICU registry (RETRAUCI)

**DOI:** 10.1186/2197-425X-3-S1-A856

**Published:** 2015-10-01

**Authors:** M Chico-Fernández, M Sánchez-Casado, F Alberdi-Odriozola, F Guerrero-López, MD Mayor-García, J Egea-Guerrero, JF Fernández-Ortega, A Bueno-González, J González-Robledo, L Servià-Goixart, J Roldán-Ramírez, MÁ Ballesteros-Sanz, E Tejerina-Álvarez, JA Llompart-Pou, GT Trauma y Neurointensivismo SEMICYUC

**Affiliations:** Hospital Universitario 12 Octubre, Madrid, Spain; Hospital Virgen de la Salud, Toledo, Spain; Hospital Universitario de Donostia, San Sebastián, Spain; Hospital Universitario Virgen de las Nieves, Granada, Spain; Complejo Hospitalario de Torrecárdenas, Almería, Spain; Hospital Universitario Virgen del Rocío, Sevilla, Spain; Hospital Universitario Carlos Haya, Málaga, Spain; Hospital General Universitario de Ciudad Real, Cuidad Real, Spain; Complejo Asistencial Universitario de Salamanca, Salamanca, Spain; Hospital Universitari Arnau de Vilanova, Lleida, Spain; Complejo Hospitalario de Pamplona, Pamplona, Spain; Hospital Universitario Marqués de Valdecilla, Santander, Spain; Hospital Universitario de Getafe, Madrid, Spain; Hospital Universitari Son Espases, Palma de Mallorca, Spain

## Introduction

Data obtained from large databases in severe trauma patients generated the opportunity to develop different models of probability of survival. These models are supported by mathematics-based severity scales. Using these models in regional areas different from those where they were developed makes its validation mandatory. The TRISS model, which combines variables of RTS, ISS, age and type of injury allows obtaining the probability of survival.

## Objectives

To validate the TRISS method as an audit system on a group of patients with severe trauma admitted to Spanish trauma intensive care units (ICUs).

## Methods

We compared the predicted mortality using the TRISS model with that observed in our sample of trauma patients admitted in 13 ICUs during 2013 and 2014, as a part of the pilot phase of the Spanish trauma ICU registry (RETRAUCI). We evaluated the discrimination of the model using receiver operating characteristic (ROC) curves and the value under its area (CI 95%) and the calibration using the Hosmer-Lemeshow (HL) goodness-of-fit test, both in patients with low and high predicted mortality. A value of p < 0.05 was considered significant.

## Results

From 2242 trauma patients in the pilot phase, data were available in 1405 patients (62.66%). Observed mortality was 18% (253 patients), while predicted mortality was 16.9%. Area under the ROC curve was 0.819 (CI 95%: 0.762-0.877; p < 0.0001). Patients with blunt trauma (1305) had an area under the ROC curve 0.819 (CI 95%: 0.759-0.879; p < 0.0001) and those with penetrating trauma (100) of 0.835 (CI 95%: 0.733-0.937; p < 0.0001). In the whole sample, the HL test showed a value of 25.382 (p = 0.001); being 27.354 (p < 0.0001) in blunt trauma and 5.907 (p = 0.658) in penetrating trauma. We observed that the TRISS model underestimated mortality in patients with low predicted mortality and overestimated mortality in patients with higher predicted mortality. The efficiency of the model, defined per percentage of patients correctly classified, was 88.04%, being slightly higher in penetrating trauma (91%).

## Conclusions

The use of the TRISS model in the attention of severe trauma in Spanish ICUs showed good levels of discrimination with an inadequate calibration, especially in blunt trauma. Penetrating trauma showed better discrimination and good calibration. Newly calibrated (b coefficient) scales are necessary in our environment.

## Grant Acknowledgment

Fundación Mutua Madrileña

